# Selective growth of ZnO nanorods on microgap electrodes and their applications in UV sensors

**DOI:** 10.1186/1556-276X-9-29

**Published:** 2014-01-15

**Authors:** Qazi Humayun, Muhammad Kashif, Uda Hashim, Ahsanulhaq Qurashi

**Affiliations:** 1 Nano Biochip Research Group, Institute of Nano Electronic Engineering (INEE), Universiti Malaysia Perlis (UniMAP), Kangar, Perlis 01000, Malaysia; 2 Center of Excellence in Nanotechnology, King Fahd University of Petroleum and Minerals Dhahran, Dhahran 31261, Saudi Arabia; 3 Department of Chemistry, King Fahd University of Petroleum and Minerals Dhahran, Dhahran 31261, Saudi Arabia

**Keywords:** Wet etching, ZnO nanorods, Microgap, Photocurrent, Photoresponse, Low-power UV sensor

## Abstract

Selective area growth of ZnO nanorods is accomplished on microgap electrodes (spacing of 6 μm) by using a facile wet chemical etching process. The growth of ZnO nanorods on a selected area of microgap electrode is carried out by hydrothermal synthesis forming *nanorod bridge* between two electrodes. This is an attractive, genuine, direct, and highly reproducible technique to grow nanowire/nanorod onto the electrodes on selected area. The ZnO nanorods were grown at 90°C on the pre-patterned electrode system without destroying the electrode surface structure interface and geometry. The ZnO nanorods were tested for their application in ultraviolet (UV) sensors. The photocurrent-to-dark (*I*ph/*I*d) ratio was 3.11. At an applied voltage of 5 V, the response and recovery time was 72 and 110 s, respectively, and the response reached 2 A/W. The deposited ZnO nanorods exhibited a UV photoresponse that is promising for future cost-effective and low-power electronic UV-sensing applications.

## Background

Metal-oxide-semiconductor nanostructures have received considerable attention worldwide because of their excellent physical and chemical properties in the recent past [1]. Among them, zinc oxide (ZnO) nanostructures have attracted significant interest because of their large wide direct bandgap (*E*g = 3.37 eV) [2] and high exciton binding energy (60 meV) [2-4]. Ultraviolet (UV) photodetectors are widely used in various commercial [5] and military applications [6], such as secure space-to-space communications [7], pollution monitoring, water sterilization, flame sensing, and early missile plume detection [8]. Moreover, the direct flow of electrons contributes to the maximum photocurrent generation because of the large interfacial surface area [9]. In contrast to GaN, ZnO has a maximum electron saturation velocity; thus, photodetectors equipped with ZnO can perform at a maximum operation speed [10]. Different types of photosensors, such as p-n junction, metal–semiconductor-metal, and Schottky diodes, have been fabricated. However, metal–semiconductor-metal photosensors are becoming popular because of their simple structure [11]. The sensor photoconductivity of ZnO depends on the growth condition, the surface morphology, and crystal quality [12].

The synthesis of ZnO nanostructures has been reported; however, the area-selective deposition of ZnO nanostructures or their integration into complex architectures (microgap electrode) is rarely reported [13-24]. In this manuscript, we report the deposition of ZnO nanorods on a selective area of microgap electrodes through simple low-cost, highly reproducible hydrothermal technique, and their applications in UV sensors were investigated.

## Methods

### Materials and method

**Figure 1:**
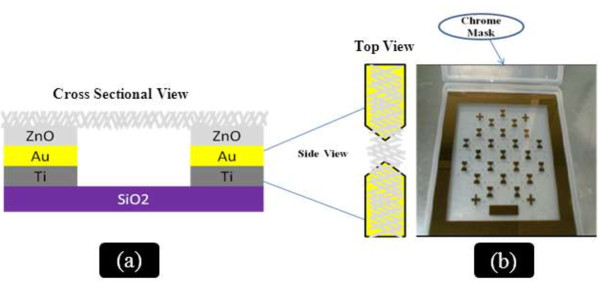
**The entire experimental process and the butterfly topology zero-gap design. (a)** Schematic of the side and top views of the entire experimental process and the **(b)** butterfly topology zero-gap design printed on the chrome mask.

**Figure 2:**
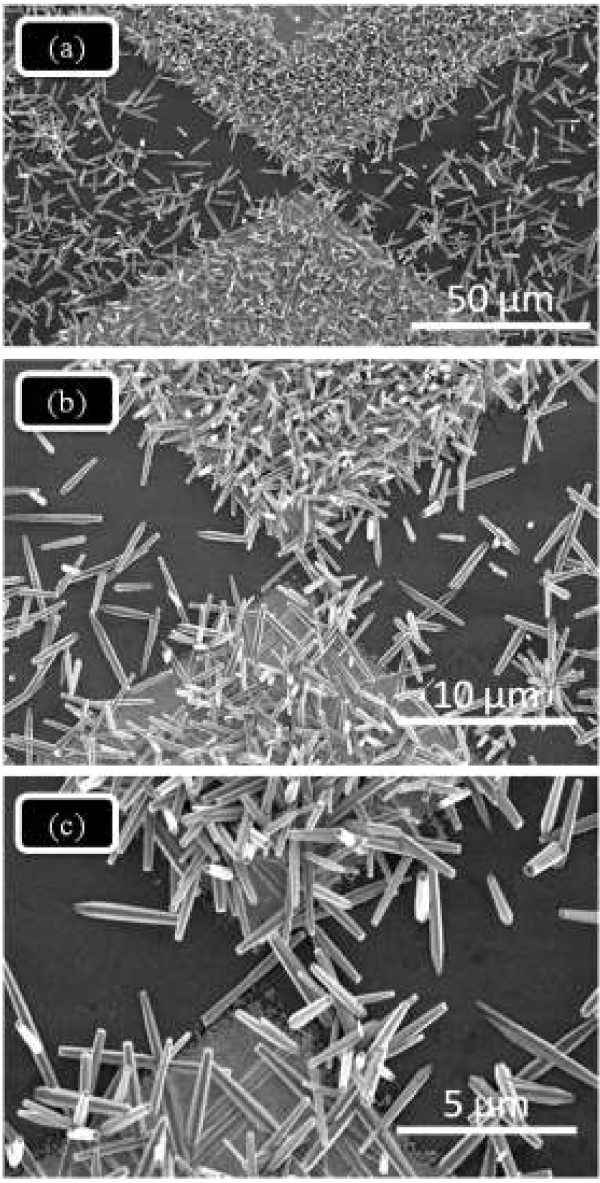
**SEM images of area-selective deposited ZnO nanorods on microgap electrodes.** The images are at different magnification powers: **(a)** 50 μm, **(b)** 10 μm, and **(c)** 5 μm.

The UV sensor was fabricated with Schottky contacts by conventional photolithography followed by wet etching technique. ZnO nanorods were grown on the electrode by hydrothermal process. The p-type (100) silicon substrate was cleaned with RCA1 and RCA2 [25] to remove the contaminants. The RCA1 solution was prepared by mixing DI water, ammonium hydroxide (NH_4_OH (27%)), and hydrogen peroxide (H_2_O_2_ (30%)) by maintaining the ratio of 5:1:1. For the RCA2 preparation, hydrochloric acid (HCL (27%)) and H_2_O_2_ (30%) were mixed in DI water by maintaining the composition at 6:1:1. An oxide layer with a thickness of approximately 1 μm was then deposited by wet oxidation process. Thin layers of titanium (Ti) (30 nm) and gold (Au) (150 nm) were deposited using a thermal evaporator. As shown in Figure 1b, a zero-gap chrome mask was used in the butterfly topology. After UV exposure, controlled resist development process was performed to obtain a 6-μm gap. The seed solution was prepared as described in our previous research [25]. The concentration of zinc acetate dehydrate was 0.35 M in 2-methoxyethanol. Monoethanolamine (MEA) was added dropwise to the seed solution, which was heated to 60°C with vigorous stirring until the molar ratio of MEA to zinc acetate dehydrate reached 1:1. The seed solution was incubated at 60°C for 2 h with continuous stirring. The measured pH value for the MEA-based seed solution was 7.69. The aged solution was dropped onto the surface of the microgap structure, which was rotated at 3,000 rpm for 45 s. After deposition via spin coating, the films were dried at 300°C for 15 min to evaporate the solvent and remove the organic residuals. The spin coating procedure was repeated five times. The films were then inserted into the furnace and annealed at 400°C for 1 h in air. The growth solution was prepared by mixing equimolar ratio zinc nitrate hexahydrate (0.025 M) and hexamethylenetetramine (0.025 M) in 150 mL of deionized (DI) water. The growth solution was transferred to a 250-mL beaker with vigorous stirring for 20 min. The pre-coated substrates were then horizontally immersed inside the beaker containing the growth precursors. The beaker was directly inserted in a preheated oven at 90°C for 6 h to induce the growth of nanorods. After the growth induction time, the oven was cooled down to room temperature. The substrate was washed with DI water to remove any residual salt and dried in nitrogen atmosphere. The aspect ratio of the ZnO nanorods depends on the reaction time. The length of the nanorods considerably increased with longer reaction times; however, the diameter of the nanorods only grew slightly. Figure 2a,b,c shows the SEM images of the ZnO nanorods at different magnification powers after 6 h of reaction time.

## Results and discussion

**Figure 3:**
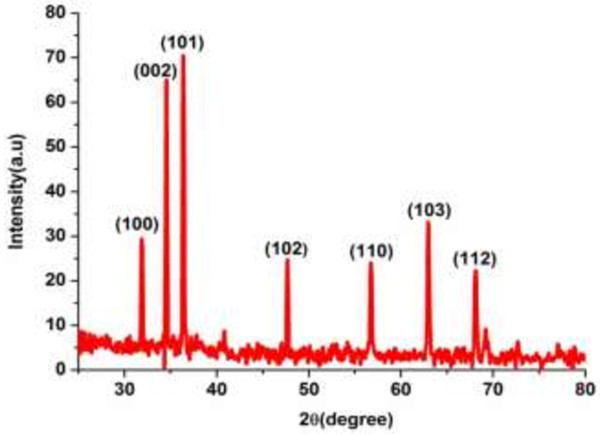
XRD spectrum of the ZnO nanorods.

The X-ray diffraction (XRD) spectrum of the ZnO nanorods calcinated at 400°C is shown in Figure 3. The peaks indicate that the nanorods have a polycrystalline phase with a preferential orientation along the *c*-axis, and that the *c*-axis of the crystalline is uniformly perpendicular to the substrate surface. The crystalline size at the (002) peak was calculated using the Scherrer formula [26-28].

Figure 1a shows the schematic view of entire experimental process. Figure 1b shows the butterfly topology zero-gap chrome mask. Figure 2a,b,c shows high- and low-magnification SEM micrographs of the deposited ZnO nanorods. The SEM showed the morphological features of the ZnO nanorods deposited on a selected area of microgap electrodes. The seeded area was completely covered with ZnO nanorods which indicates selective growth on the area of microgap electrodes. It is noteworthy to mention that the as-grown ZnO nanorods were interconnected to each other as noticeably seen by the SEM observations [29-31]. Such interconnected network facilitates electron transport along the nanorod/nanowire axis [32,33].

**Figure 4:**
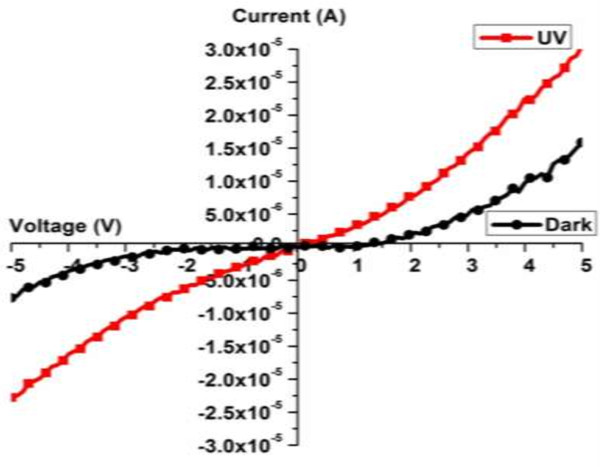
***I*-*V* curves of the area-selective deposited ZnO nanorods in dark and UV light environments.**

Figure 4 demonstrates the current-to-voltage (*I*-*V*) characterization of the area-selective deposited ZnO nanorods on the microgap electrodes. These *I*-*V* values were recorded in the dark and with UV illumination. The *I*-*V* curves show the Schottky behavior of Au on an n-type ZnO contact. Such behavior corresponds to the large leakage resistance and high quality of the contacts [34]. The dark and photocurrent values were 7.35 and 22.89 μA, respectively, which clearly indicate a threefold increase in the dark current value.

The sensor mechanism is based on Equations (1) to (3) [35,36]; the reactions on the ZnO nanorod surface during UV illumination can be explained as follows: when the adsorbed oxygen molecules capture the electron from the conduction band, a negative space charge layer is created, which results in enhanced resistivity [37].

(1) $$O_{2}+e^{-}\to O_{2}^{-}.$$

When the photon energy is greater than the bandgap energy (*E*g), the incident radiation is adsorbed in the ZnO nanorod UV sensor, which results in electron–hole pairs.

(2) $$\mathrm{hv}\to h^{+}+e^{-}.$$

The positively charge holes that were created due to the photogeneration neutralize the chemisorbed oxygen that was responsible for higher resistance that revealed conductivity increment, and as a consequence, the photocurrent increases.

$$O_{2}^{-}+h^{+}\to O_{2},$$

where O_2_ is the oxygen molecule, *e*^-^ is the free electron and the photogenerated electron in the conduction band, $O_{2}^{-}$ is the adsorbed oxygen, *hv* is the photon energy of the UV light, and *h*^+^ is the photogenerated hole in the valence band. After the UV light is switched on, the number of oxygen molecules on the ZnO nanorod surface rapidly reaches the maximum value in response to the ultraviolet light [38]. When the ultraviolet light is switched off, the oxygen molecules are reabsorbed on the ZnO nanorod surface. Thus, the sensor reverts to its initial mode [39].

An important parameter used to evaluate the suitability of the sensor for UV-sensing applications is spectral responsivity as a function of different wavelengths. This parameter yields the internal photoconductive gain.

**Figure 5:**
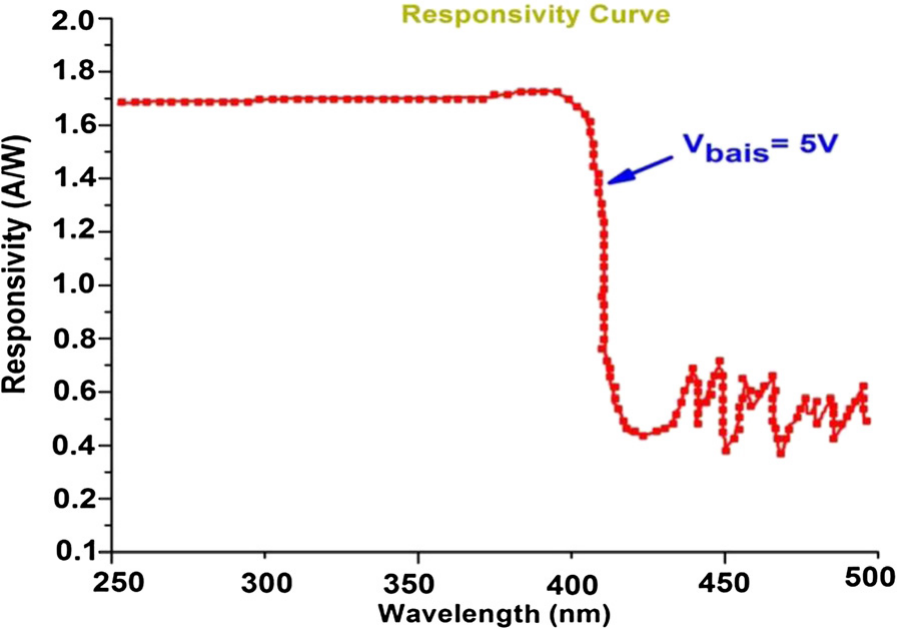
Spectral responsivity of area-selective deposited ZnO nanorods between the microgap electrodes.

Generally, the sensor responsivity can be calculated as [40]

(4) $$R_{i}=\mathrm{\eta g}\frac{\mathrm{q\lambda}}{\mathrm{hc}},$$

where *λ*, *q*, *h*, *c*, and *η* show the wavelength, electron charge, Planck's constant, light velocity, external quantum efficiency, and internal gain of the sensor. As shown in Figure 5, the sensor responsivity shows a linear behavior below the bandgap UV region (300 to 370 nm) and a sharp cutoff with a decrease of two to three orders of magnitude at approximately 370 nm. The maximum responsivity of our sensor at an applied bias of 5 V was 2 A/W, which is higher than the values reported in the literature [41-43].

**Figure 6:**
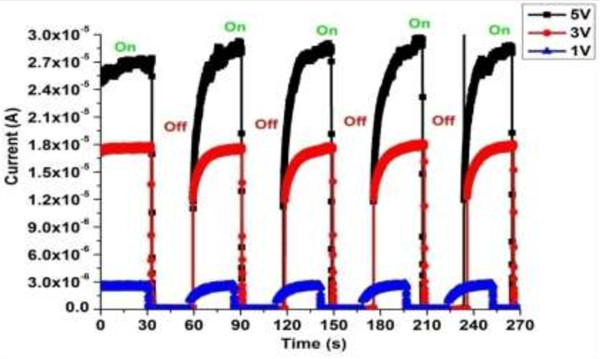
***I*-*t* curve of the area-selective deposited ZnO nanorods in dark and UV light environments.**

Another important parameter for UV sensor is the current-to-time (*I*-*t*) response in the switched on/off states of UV light. Figure 6 shows the *I*-*t* response curves at different voltages of area-selective deposited ZnO nanorods on microgap electrodes with UV illumination. The rise time was 72 s, whereas the decay time was 110 s. We believe that such rise and decay times observed in our photo response measurement are caused by area-selective deposited ZnO nanorods on the microgap electrodes. Also from the curves, it can be revealed that the fabricated devices can be used for low-power miniaturized devices with fast detection capability and reproducibility.

## Conclusions

In summary, the ZnO nanorods were selectively grown on pre-patterned seeded substrates at low temperature (90°C) by hydrothermal method. Conventional lithography followed by simple wet etching process was used to define microgap electrodes with approximate spacing of 6 μm on seeded substrates. The ZnO nanorod microgap electrodes were investigated in dark and UV environments and showed noticeable changes with UV light exposure. The sensor gain was 3.11. The response time was less than 72 s. The recovery time was 110 s. The responsivity was 2 A/W. These fascinating results propose that the selective area growth of the ZnO nanorods exhibits a UV photoresponse that is promising for future cost-effective and low-power electronic UV-sensor applications.

## Competing interests

The authors declare that they have no competing interests.

## Authors' contributions

QH and MK carried out the synthesis, characterization, and the sensing study of the nanorods. AQ provided technical writing support on the manuscript. UH provided all the instruments used for characterization. All authors read and approved the final manuscript.

## Authors' information

QH is a PhD Student at the Institute of Nano Electronic Engineering University Malaysia Perlis. MK is a Post Doctorate Fellow at the Institute of Nano Electronic Engineering University Malaysia Perlis. UH is a Professor and Director of the Institute of Nano Electronic Engineering University Malaysia Perlis. AQ is an Assistant Professor at the Center of Excellence in Nanotechnology and Chemistry Department of King Fahd University of Petroleum and Minerals, Saudi Arabia.
